# Knowledge of Acute Rheumatic Fever and Rheumatic Heart Disease Prevention Among House Officers in Three Teaching Hospitals, Khartoum, Sudan

**DOI:** 10.7759/cureus.97424

**Published:** 2025-11-21

**Authors:** Hussein J Ahmed, Othman Abdelrahman

**Affiliations:** 1 Surgery, University of Khartoum, Khartoum, SDN; 2 Surgery, Faculty of Medicine, University of Khartoum, Khartoum, SDN

**Keywords:** acute rheumatic fever, knowledge, prevention, rheumatic heart disease, sudan, sudan khartoum

## Abstract

Background: Acute rheumatic fever (ARF) and rheumatic heart disease (RHD) remain major causes of cardiovascular morbidity and mortality in Sudan. House officers are often the first point of contact in hospitals, making their knowledge critical for effective prevention. This study assessed the knowledge of house officers regarding the prevention of ARF and RHD.

Methods: A cross-sectional study was conducted from October 2017 to February 2018 at three major teaching hospitals (Omdurman, Bahri, and Ibrahim Malik) in Khartoum. A sample of 186 house officers was recruited. Data were collected using a self-administered questionnaire to assess knowledge of Group A streptococcal (GAS) pharyngitis diagnosis, ARF diagnosis using the 2015 Jones criteria, and primary and secondary prevention strategies. Data were analyzed using SPSS version 20 (IBM Corp., Armonk, NY).

Results: The response rate was 100% (186 participants), comprising 47.3% males and 52.7% females. Knowledge of the clinical diagnosis of GAS pharyngitis per national guidelines was low (30%). Knowledge of the first-line drug for treatment was 44%, while awareness of the second-line drug was higher (64%). The understanding of ARF diagnosis for a new case was 68.8%, but only 43.5% for a recurrent case. Knowledge of primary prevention was 79%, while secondary prevention was 53.8%. Awareness of the correct duration of secondary prophylaxis was 30% for cases without carditis and 55.9% for cases with carditis. Overall, 17.7% of participants had poor knowledge (≤31% correct answers), 64% had average knowledge (37%-69% correct), and 17.7% had sufficient knowledge (≥75% correct).

Conclusion: The overall knowledge of house officers regarding ARF and RHD prevention was average. Significant gaps were identified in the diagnosis of GAS pharyngitis, management of recurrent ARF, and protocols for secondary prophylaxis. Targeted educational interventions are urgently needed to improve adherence to national guidelines.

## Introduction

Acute rheumatic fever (ARF) is a multisystem inflammatory disease and a delayed sequela of Group A streptococcal (GAS) pharyngitis, primarily affecting the joints, heart, and brain [1]. The most significant complication is rheumatic heart disease (RHD), characterized by permanent damage to the heart valves, which can lead to heart failure, stroke, and premature death [2]. Despite being preventable, RHD remains a devastating public health problem in low- and middle-income countries. However, globally, it affects over 33 million people and causes an estimated 275,000 deaths annually [3,4]. Sudan bears a particularly high burden, with a reported RHD prevalence of 10.2 per 1000, significantly higher than neighboring countries [5]. Many patients present late with severe valve disease requiring surgery, access to which is severely limited [6,7]. The cornerstone of controlling this disease is a robust strategy of primary prevention (timely treatment of GAS pharyngitis to prevent initial ARF episodes) and secondary prevention (continuous antibiotic prophylaxis to prevent recurrent ARF and disease progression) [8]. In 2012, Sudan adopted a new national program based on Awareness, Surveillance, Advocacy, and Prevention (ASAP) to standardize this approach [9]. House officers (intern doctors) form the frontline of hospital care and play a pivotal role in the initial diagnosis, management, and patient education regarding ARF/RHD. Their knowledge is therefore fundamental to the success of national control efforts. This study aimed to assess the knowledge of house officers regarding the prevention of ARF and RHD in teaching hospitals in Sudan, according to the 2015 revised Jones criteria and the Sudan national program guidelines. Understanding current knowledge levels among frontline physicians is essential to identify educational gaps and to inform targeted interventions that can ultimately improve adherence to Sudan national protocols and enhance long-term patient outcomes.

## Materials and methods

Study design and setting

This descriptive, facility-based, cross-sectional study was conducted from October 2017 to February 2018 at Omdurman Teaching Hospital, Bahri Teaching Hospital, and Ibrahim Malik Teaching Hospital in Khartoum, Sudan.

Study population and sampling

All house officers working at the three hospitals during the study period were eligible. The total population was 348. A sample size of 186 was calculated using the formula n = N/(1 + Ne²), with e = 0.05. A non-probability, convenience sampling technique was employed. All eligible house officers who were on duty and consented to participate at the time of data collection were included until the desired sample size was reached.

Data collection tool and technique

Certainly, here is a concise, polished, and cohesive version of your methods section that preserves all essential details while improving flow, clarity, and academic tone.

Data collection

Data were collected through the direct, in-person distribution of a structured, self-administered questionnaire. Trained data collectors hand-delivered the questionnaires to house officers during working hours in each participating hospital. Participants completed the forms independently and returned them anonymously to sealed collection boxes placed in their departments.

To minimize response and selection bias, all eligible house officers present during the study period were invited to participate voluntarily, and no identifying information (such as names or staff numbers) was collected. Data collectors provided clarification only upon request to avoid influencing responses, thereby ensuring anonymity and confidentiality.

The questionnaire was structured, closed-ended, and designed to assess knowledge across four key domains: (1) Diagnosis of GAS pharyngitis according to the Sudan national ARF/RHD prevention program (ASAP). (2) Treatment of GAS infection, including the appropriate antibiotic choice, dose, and duration. (3) Diagnosis of ARF based on the 2015 revised Jones Criteria, requiring evidence of preceding streptococcal infection plus either two major or one major and two minor criteria (major: carditis, polyarthritis, chorea, erythema marginatum, and subcutaneous nodules; minor: fever, arthralgia, elevated ESR or CRP, and prolonged PR interval). (4) Primary and secondary prevention strategies, focusing on antibiotic prophylaxis regimens and the recommended duration of therapy.

Ethical considerations

The study was approved in 2018 by the Institutional Review Board of Community Medicine, University of Khartoum, Faculty of Medicine. Written informed consent was obtained from all participants.

Data analysis

Data were coded and entered into SPSS Statistics version 20 (IBM Corp., Armonk, NY). Descriptive statistics were expressed as frequencies and percentages. Inferential analyses (chi-square test, Student’s t-test, or ANOVA as appropriate) were performed, and results are presented with test statistics (χ², t, or F) and corresponding p-values. Statistical significance was set at p < 0.05. The cut-off points used to categorize knowledge levels were defined as follows: Poor knowledge: ≤31% correct answers, average knowledge: 37%-69% correct answers, and sufficient knowledge: ≥ 75% correct answers.

## Results

**Figure 1:**
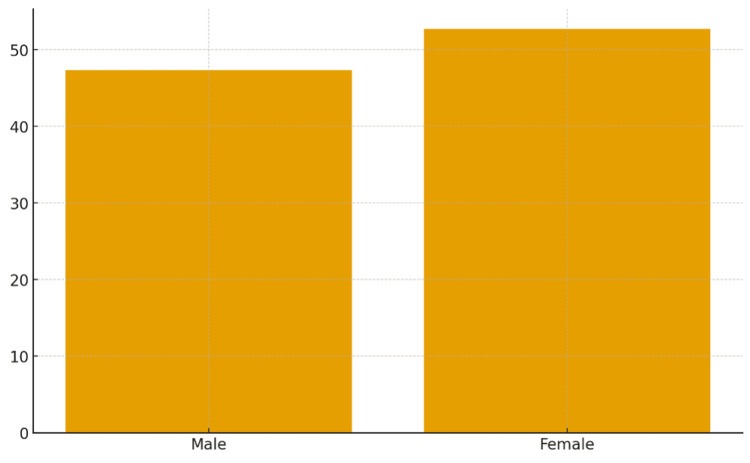
Proportional representation of male and female participants

A total of 186 house officers participated (response rate 100%). Of these, 88 (47.3%) were male and 98 (52.7%) were female (Table 1, Figure 1). Most participants (162, 87%) reported that their main source of knowledge was the medical school curriculum, while only 4 (2.2%) had attended formal training workshops.

**Table 1:** Gender distribution of participants

Gender	N	%
Male	88	47.3
Female	98	52.7

Awareness of GAS pharyngitis

Only 56 participants (30%) correctly identified the clinical diagnosis of GAS pharyngitis according to the national program.

Knowledge of treatment

**Figure 2:**
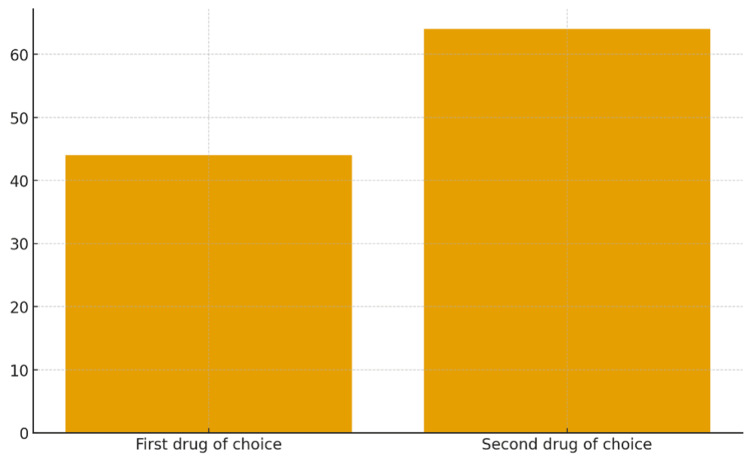
Correct identification of first- and second-line antibiotics for GAS pharyngitis GAS, Group A streptococcal.

Eighty-two (44%) participants identified benzathine penicillin as the first-line treatment, while 119 (64%) correctly recognized erythromycin as the second-line option for penicillin-allergic patients (Figure 2, Table 2).

**Table 2:** Participants’ knowledge of recommended antibiotic choices for GAS pharyngitis GAS, Group A streptococcal.

Treatment knowledge	N	%
First drug of choice (benzathine penicillin)	82	44
Second drug of choice (erythromycin)	119	64

Knowledge of ARF diagnosis

**Figure 3:**
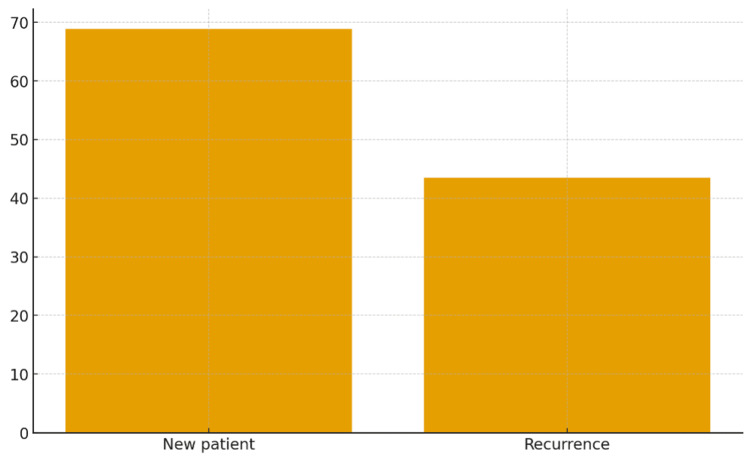
Proportion of participants accurately diagnosing new and recurrent ARF cases ARF, acute rheumatic fever.

Knowledge of Jones criteria varied: 128 (68.8%) participants correctly identified features of a new ARF case, but only 81 (43.5%) did so for recurrent cases (Figure 3, Table 3). Primary criteria awareness was highest for monoarthritis (106, 56.8%) and lowest for elevated erythrocyte sedimentation rate (ESR) (15, 8%). For minor criteria, 101 (54.1%) participants identified monoarthralgia, while only 5 (2.4%) misidentified monoarthritis. Regarding sufficient evidence of ARF, 112 (60.3%) participants recognized Sydenham chorea, while only 13 (6.9%) identified subcutaneous nodules. 

**Table 3:** Accuracy of participants’ knowledge regarding ARF diagnostic criteria ARF, acute rheumatic fever.

ARF diagnosis	N	%
New patient	128	68.8
Recurrence	81	43.5

Knowledge of prevention

**Figure 4:**
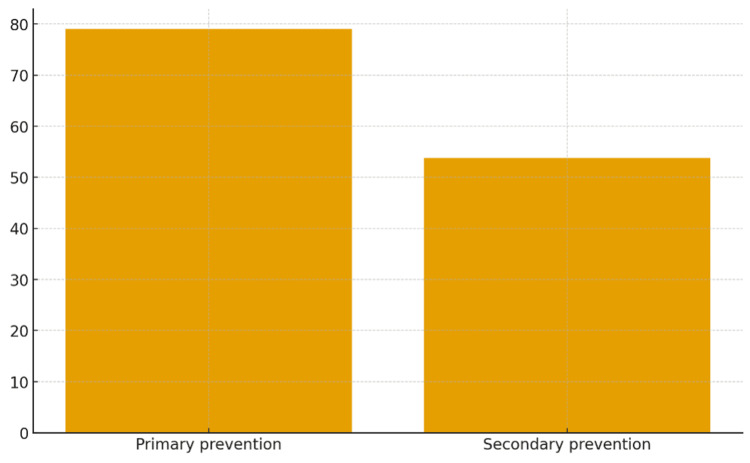
Proportion of participants with knowledge of primary and secondary prevention of ARF and RHD ARF, acute rheumatic fever; RHD, rheumatic heart disease.

**Figure 5:**
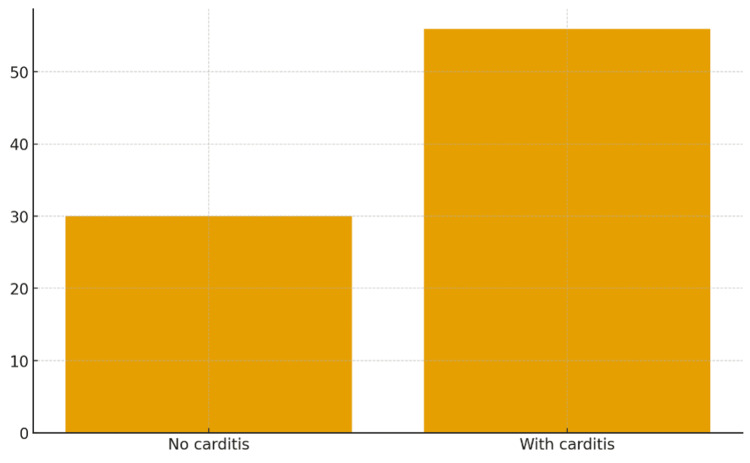
Knowledge about duration of secondary prevention

Knowledge of primary prevention was relatively good (147, 79%), whereas secondary prevention knowledge was lower (100, 53.8%) (Table 4, Figure 4). Only 56 (30%) participants knew the correct prophylaxis duration for cases without carditis, compared to 104 (55.9%) for cases with carditis (Figure 5).

**Table 4:** Distribution of participants’ knowledge in primary and secondary prevention of ARF and RHD ARF, acute rheumatic fever; RHD, rheumatic heart disease.

Prevention type	N	%
Primary prevention	147	79
Secondary prevention	100	53.8

Overall knowledge levels

**Figure 6:**
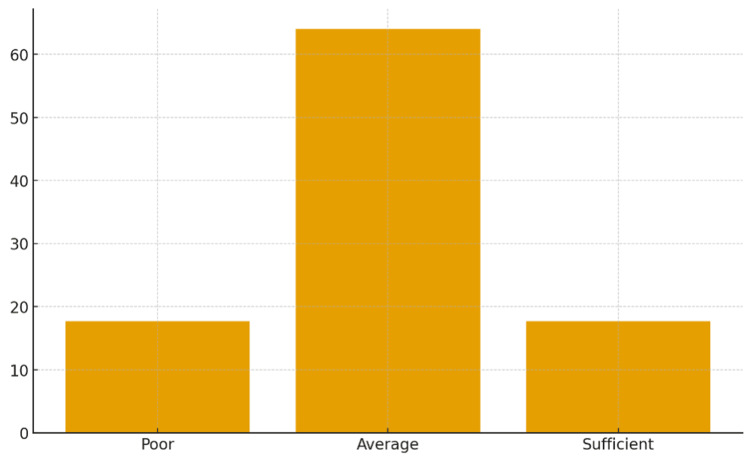
Overall categorization of participants’ total knowledge levels

Based on scoring, 33 (17.7%) participants had poor knowledge, 120 (64.6%) had average knowledge, and 33 (17.7%) had sufficient knowledge (Figure 6).

## Discussion

This study reveals that the knowledge of house officers in Khartoum regarding the prevention of ARF and RHD is suboptimal when compared with the standards outlined in the Sudan national ASAP program and the 2015 revised Jones criteria for diagnosis of ARF [1,9]. Each domain of knowledge demonstrates specific deficiencies that warrant targeted educational interventions.

Awareness and diagnosis of GAS pharyngitis

Only 30% of participants correctly identified the clinical diagnostic features of GAS pharyngitis. According to national and WHO guidelines, in resource-limited and endemic settings, diagnosis should rely primarily on clinical criteria rather than laboratory confirmation, focusing on sore throat with exudative tonsils, tender anterior cervical lymphadenopathy, fever, and absence of cough or rhinorrhea [5,9]. The low awareness observed suggests that many house officers remain dependent on laboratory investigations, contrary to local recommendations that emphasize prompt clinical diagnosis and empirical treatment to prevent the first episode of ARF [9].

Knowledge of first- and second-line treatment

Recognition of benzathine penicillin as the first-line drug for treating GAS pharyngitis was only 44%, while 64% correctly identified erythromycin as the second-line option for penicillin-allergic patients. This contrasts with the national ASAP guidelines and WHO recommendations, which specify benzathine penicillin as the preferred first-line agent in endemic countries [5,9]. The discrepancy likely reflects outdated teaching materials and discomfort with intramuscular administration due to fear of anaphylaxis. Training should reinforce that a skin test is unnecessary, provided adrenaline is available, and lidocaine may be used to reduce injection pain [5,9].

Knowledge of ARF diagnosis: new vs. recurrent cases

Approximately 68.8% of respondents correctly identified diagnostic criteria for a new case of ARF, but only 43.5% did so for a recurrent case. The 2015 revised Jones criteria recognize that, in high-risk populations such as Sudan, monoarthritis and polyarthralgia may serve as major manifestations, and subclinical carditis should be considered a major criterion [1]. Recurrent cases require only one major or two minor criteria plus evidence of recent streptococcal infection. The decline in knowledge for recurrent cases is concerning, as recurrent ARF is the main driver of progressive valve damage and chronic RHD [2,3]. These findings are consistent with Khalid et al., who reported similar deficiencies among Sudanese physicians [7].

Understanding of major and minor Jones criteria

Participants demonstrated confusion between monoarthritis (a major criterion in high-risk settings) and monoarthralgia (a minor criterion). Additionally, a few recognized elevated ESR or prolonged PR interval as minor criteria. Proper application of the Jones criteria is critical for early identification of ARF and prevention of recurrence [1,9]. The findings underscore the need for structured clinical training using case-based learning and echocardiographic examples to reinforce diagnostic accuracy.

Knowledge of primary and secondary prevention

Knowledge of primary prevention (79%) was higher than that of secondary prevention (53.8%). The national ASAP program highlights both, but secondary prophylaxis, regular three-weekly benzathine penicillin injections, is the most effective strategy to prevent recurrence and disease progression [5,9]. The lower awareness of secondary prophylaxis mirrors findings from Tanzania and other low-income countries [10]. This gap implies insufficient emphasis on long-term follow-up and patient counseling in undergraduate and early postgraduate curricula.

Duration of secondary prophylaxis

Only 30% of respondents knew the correct prophylaxis duration for cases without carditis, and 55.9% for cases with carditis. According to the Sudan national RHD control program and WHO guidelines, secondary prophylaxis should continue for at least five years or until age 21 years for cases without carditis, and 10 years or until age 40 years (or lifelong) for cases with persistent valvular disease [5,9]. Misunderstanding these timelines can lead to premature cessation of prophylaxis and higher recurrence risk. Structured teaching modules and discharge checklists could improve adherence to these standards.

Overall knowledge level

Overall, 17.7% of house officers demonstrated poor knowledge (≤31%), 64.6% had average knowledge (37%-69%), and only 17.7% achieved sufficient knowledge (≥75%). Although this is consistent with earlier Sudanese studies [11], it remains below the competence level expected of first-line physicians managing ARF/RHD patients. The limited participation in formal training workshops (2.2%) highlights the lack of continuous professional development activities. Regular, mandatory continuing medical education workshops could address these gaps.

To bridge the gap between the observed and expected levels of knowledge, several measures should be implemented. Recurrent ARF recognition should be integrated into clinical training through structured case-based discussions and scenario-based learning. The correct use of benzathine penicillin must be reinforced through practical workshops that focus on safe administration techniques, management of allergic reactions, and methods to reduce injection pain. Documentation of prophylaxis duration should be standardized within ward discharge summaries and electronic health record systems to ensure uniform adherence to national guidelines. Educational priorities also need to be rebalanced to provide equal emphasis on both primary and secondary prevention strategies during undergraduate and internship training. Furthermore, regular audit and feedback mechanisms should be established in healthcare facilities to monitor compliance with ARF and RHD prevention protocols and to promote continuous quality improvement. Targeted education and regular workshops are urgently needed to strengthen adherence to guidelines and improve outcomes.

Strengths and limitations

This study has several notable strengths. It provides important baseline data on the knowledge of house officers regarding the prevention of ARF and RHD in Sudan, an area where limited data are available. The study was conducted across three major teaching hospitals in Khartoum, ensuring that the findings are representative of a broad training environment. Moreover, the 100% response rate enhances the reliability of the data and minimizes the risk of non-response bias. The use of a structured, self-administered questionnaire based on the national ASAP programme and the 2015 revised Jones criteria strengthens the validity of the findings. However, some limitations should be acknowledged. The study was limited to hospitals in Khartoum and may not reflect the situation in other regions of Sudan. Despite these limitations, the findings provide a valuable foundation for future educational interventions aimed at improving ARF and RHD prevention practices among junior doctors.

## Conclusions

Although the overall knowledge among house officers was classified as “average,” it remains below the national expectations. Focused educational interventions aligned with the ASAP program are urgently required to strengthen early diagnosis, appropriate antibiotic therapy, and sustained secondary prophylaxis, thereby improving RHD prevention outcomes in Sudan.

## Appendices

**Table 5:** Collected data

Section / Question	Options / Response Choices
Consent	Do you agree to fill this questionnaire? 1. Yes 2. No
If Yes, please continue:	
1. Age	__________
2. Gender	1. Male 2. Female
3. Current working place	1. Omdurman Hospital 2. Bahri Teaching Hospital 3. Ibrahim Malik Teaching Hospital 4. Other, specifiy ..........
4. From 1 to 5: How do you rate your level of information about prevention of ARF and RHD? (1 = highest, 5 = lowest)	1. One 2. Two 3. Three 4. Four 5. Five
5. From which sources did you acquire MOST of your information about prevention of ARF and RHD?	1. Faculty curriculum 2. Internet, publications, journals 3. Training workshops 4. Other (please specify): __________
6. The best way to diagnose bacterial pharyngitis (BPh) in patients aged 3–18 years in endemic areas is by:	1. Clinical findings 2. Throat culture and rapid antigen test 3. High leukocyte count 4. High ESR 5. I don’t know
7. Clinical findings of bacterial pharyngitis include:	1. Sore throat with exudates and pus at pharynx 2. Sore throat with absence of runny nose and cough 3. Sore throat with anterior cervical lymphadenitis 4. Sore throat with congested confluent tonsils 5. I don’t know
8. Which sign favors bacterial over viral pharyngitis?	1. Hoarse voice 2. High ASO titre 3. Cough 4. Fever 37.8°C 5. Absence of runny nose 6. I don’t know
9. Drug of choice for treatment of bacterial pharyngitis:	1. Oral penicillin 2. Oral amoxicillin 3. Azithromycin 4. One shot of benzathine penicillin 5. Procaine penicillin 6. I don’t know
10. Second drug of choice in hypersensitivity to first drug of choice:	1. Erythromycin 2. Azithromycin 3. Sulphonamide 4. Clarithromycin 5. I don’t know
11. When giving benzathine penicillin:	1. Skin test should NOT be done 2. You need to have adrenaline 3. Lidocaine is used to decrease pain 4. All the above 5. I don’t know
12. Diagnosis of ARF in new patients includes:	1. One major + high ASO 2. Two minor + high ASO 3. Subclinical carditis + high ASO 4. Two major + high ASO 5. I don’t know
13. Diagnosis of recurrence of ARF is made with:	1. One major or two minor + high ASO 2. Same as new episode 3. Carditis without other criteria 4. Fever with high ASO 5. I don’t know
14. Which is NOT a major criterion?	1. Carditis 2. Subclinical carditis 3. Prolonged PR interval 4. Monoarthritis 5. I don’t know
15. Which is a minor criterion of RF?	1. Monoarthritis 2. Polyarthralgia 3. Skin nodules 4. Monoarthralgia 5. I don’t know
16. Which is enough evidence of ARF?	1. Carditis 2. Sydenham chorea 3. Subcutaneous nodules 4. Migratory arthritis 5. I don’t know
17. Which is NOT a feature of rheumatic carditis?	1. Pansystolic apical murmur 2. Early diastolic murmur with large volume pulse 3. Heart failure without murmurs 4. Large volume pulse with Corrigan’s sign 5. I don’t know
18. Treatment of ARF includes all except:	1. High-dose aspirin 2. Bed rest 3. Benzathine penicillin 4. Steroids in all patients with carditis 5. I don’t know
19. Primary prevention of ARF means:	1. Prompt diagnosis and treatment of bacterial pharyngitis 2. 3-weekly penicillin 3. Diagnosis and treatment of ARF 4. Management of RHD 5. I don’t know
20. True or false: Tonsillectomy prevents occurrence of ARF.	1. True 2. False 3. I don’t know
21. Secondary prophylaxis of ARF means:	1. Giving 3-weekly benzathine penicillin to prevent recurrence 2. Benzathine penicillin to all with high ASO 3. Aspirin 75 mg/kg/day for 4 weeks 4. Single injection of benzathine penicillin for sore throat 5. I don’t know
22. Duration of secondary prevention if no carditis:	1. Up to 5 years 2. Up to 10 years 3. Up to 25 years 4. For life 5. I don’t know
23. Duration of secondary prevention if there is carditis:	1. Up to 5 years 2. Up to 10 years 3. Up to 25 years 4. For life 5. I don’t know
24. Which is FALSE regarding tertiary prevention of RHD?	1. Includes anti–heart failure medications 2. Recurrence may occur despite adherence to prophylaxis 3. Warfarin should be limited after valve surgery 4. Continuous benzathine penicillin after valve surgery 5. I don’t know

## References

[1] Tandon R (2012). Rheumatic fever pathogenesis: approach in research needs change. Ann Pediatr Cardiol.

[2] (2025). World Health Organization: Cardiovascular diseases (CVDs) - key facts. https://www.who.int/news-room/fact-sheets/detail/cardiovascular-diseases-(cvds).

[3] Carapetis JR, Steer AC, Mulholland EK, Weber M (2005). The global burden of group A streptococcal diseases. Lancet Infect Dis.

[4] Zühlke LJ, Steer AC (2013). Estimates of the global burden of rheumatic heart disease. Glob Heart.

[5] (2025). Rheumatic fever and rheumatic heart disease : report of a WHO Expert Consultation, Geneva, 29 October - 1 November, 2001. https://catalog.nlm.nih.gov/discovery/fulldisplay/alma9915312103406676/01NLM_INST:01NLM_INST.

[6] Labarthe DR (1999). Prevention of cardiovascular risk factors in the first place. Prev Med.

[7] Khalid S, Ali M, Eldaim IN, Osman SH, Bakhite SM (2012). Clinical and echocardiographic features of children with rheumatic heart disease and their serum cytokine profile letter to the editors. Pan Afr Med J.

[8] Karthikeyan G, Watkins D, Bukhman G (2023). Research priorities for the secondary prevention and management of acute rheumatic fever and rheumatic heart disease: a National Heart, Lung, and Blood Institute workshop report. BMJ Glob Health.

[9] Tastan A, Ozturk A, Senarslan O, Ozel E, Uyar S, Ozcan EE, Kozan O (2016). Comparison of two different techniques for balloon sizing in percutaneous mitral balloon valvuloplasty: which is preferable?. Cardiovasc J Afr.

[10] María MR (2025). Awareness of rheumatic heart disease prevention among primary health care providers and people aged nine years and above in Kinondoni municipality Dar es salaam, Tanzania.

[11] Adam MH, Ali AG, Farah AM (2023). Diagnosis and management of acute pharyngotonsillitis among pediatric patients at Ribat Teaching Hospital: a prospective audit (2021-2022). Sudan J Paediatr.

